# Post-traumatic growth and its influencing factors in first-episode stroke patients: a cross-sectional study

**DOI:** 10.1186/s40359-025-03347-y

**Published:** 2025-08-27

**Authors:** Minli Hu, Yue Ban, Zhihui Li, Yu He, Liping Deng, Xiaohua Xie

**Affiliations:** 1 School of Nursing, Anhui Medical University, Hefei, 230032 China; 2 Department of Nursing, The First Affiliated Hospital of Shenzhen University, Shenzhen Second People’s Hospital, Shenzhen, 518035 China

**Keywords:** Post-traumatic growth, Stroke, Coping strategies, Social support

## Abstract

**Purpose:**

The purpose of this study was to describe the level of posttraumatic growth in first-time stroke patients and to explore its impact on posttraumatic growth.

**Method:**

A cross-sectional survey with a convenience sampling method was employed to collect data using the Post-traumatic Growth Inventory (PTGI), Social Support Rating Scale (SSRS), Brief Event-Related Reflection Questionnaire (C-ERRI), Simplified Coping Style Questionnaire (SCSQ), Generalized Anxiety Disorder Self-Assessment Scale (GAD-7), and Patient Health Questionnaire Depression Symptom Checklist (PHQ-9). From August 2024 to February 2025,166 participants were recruited from a hospital in Shenzhen, Guangdong Province, to complete the questionnaires.

**Results:**

PTG was present in Chinese first stroke patients with a mean PTGI score of (61.03 ± 13.92). In the Post-traumatic Growth Inventory (PTGI), the dimension with the highest percentile score was “Relating to Others,” while the lowest was “New Possibilities.” Significant predictors of post-traumatic growth included positive coping, negative coping, age, and personality traits.

**Conclusions:**

The findings of this study indicate that patients experiencing their first stroke who employ positive coping and exhibit extroverted personality traits are more likely to achieve significant posttraumatic growth. In contrast, an excessive reliance on negative coping can adversely affect the facilitation of PTG. It is recommended that personalized intervention measures be provided for introverted first-time stroke patients, with the aim of promoting PTG, assisting them in better adapting to the challenges associated with the disease, and enhancing their quality of life.

**Clinical trial number:**

Not applicable.

**Supplementary Information:**

The online version contains supplementary material available at 10.1186/s40359-025-03347-y.

## Introduction

Stroke, also known as cerebrovascular accident (CVA), represents a category of diseases with high disability and mortality rates both in China and globally [1].According to the Global Burden of Disease (GBD) report, the incidence of stroke in China is the highest in the world, about 39.9% [2], and the number of new stroke patients in China can be up to 2.4 million per year, which is still on the rise [3]A patient’s first stroke, which results in a sudden change from a healthy state to a disabled state and a sudden change in mental life, socialization, and social status, can cause the individual to suffer a great psychological burden [4].Currently, There is a growing body of research on positive psychological correlates of stroke patients, such as well-being, post-traumatic growth, hope, self-efficacy, etc. With the emergence and rapid development of positive psychology, the research perspectives and scopes of scholars have been further expanded and transformed. People are no longer solely relying on the traditional psychological concepts of negative psychology to identify patients’ psychological issues and seek ways to repair their negative mental states. Instead, researchers are now committed to discovering and deeply exploring the potential and latent abilities of each patient. By uncovering their positive motivation and strengths, patients are encouraged to face traumatic life events with a positive attitude. This approach not only promotes mental and physical health but also enhances the overall quality of life.

Post-traumatic growth (PTG) refers to the positive changes that occur in individuals after experiencing various traumas [5]. It manifests itself in a variety of ways, including increased appreciation of life, more meaningful relationships, increased sense of personal power, changed priorities, and a richer existential and spiritual life [6]. As early as 1991, Thompson [7]et al. found that stroke patients experienced some positive changes after the onset of the disease. By 2005, Gillen [8]et al. found through qualitative interviews that 10 out of 16 stroke patients were able to perceive positive emotions. Positive psychology research on the disease is currently receiving a lot of attention both nationally and internationally.

Coping strategies is a term that refers to both a patient’s internal activities as well as his or her external communications and behaviors aimed at alleviating the pain and suffering caused by the illness [9]. As the technical means by which patients cope with their illness, coping strategies reflect both their usual style of coping and their efforts to try new approaches to the specific challenges posed by their illness [9].The results of the study suggest that brain-injured patients who employ positive and adaptive coping strategies tend to have a more significant likelihood and degree of subsequent post-traumatic growth [10].There are two main types of coping strategies: positive and negative coping. Positive coping is having the ability to solve problems, which helps people cope with the source of stress more quickly and better deal with the reactions that come with stress. In contrast, negative coping is often detrimental to mental health. Both types of coping are important bridges between stress and mental health [11]. 

Rumination was proposed by Tedeschi and Calhoun in 2004 [6], based on the theory of post-traumatic growth. It refers to the cognitive processing of event-related information after an individual has experienced a traumatic event or a negative change. Rumination is a key link in the realization of post-traumatic growth and includes two types: deliberate rumination and intrusive rumination. Deliberate rumination refers to the process of actively trying to understand the event, seeking solutions to problems, and constructing meaning from the experience. This adaptive form of cognitive processing helps individuals make sense of their trauma and find ways to move forward. In contrast, intrusive rumination involves unwanted, repetitive, and negative thinking that arises from repeated, passive attention to a traumatic event. This non-adaptive form of cognitive processing can exacerbate distress and hinder recovery. Current research on stroke patients shows that purposeful rumination is positively associated with PTG and that rumination mediates the relationship between social support and post-traumatic growth [12].

Social support is defined as information that leads individuals to believe that they are cared for and loved, respected, and part of a network of mutual obligations [13]. According to Tedeschi and Calhoun’s research [6], the social support received by an individual is a significant predictor of the ability to achieve posttraumatic growth after a traumatic event. Measures of social support come from different constructs, including received support, perceived support, and satisfaction with social support. Studies have confirmed that social support can alleviate depressive symptoms during the course of stroke patients’ illness, improve quality of life, and have a positive effect on mental health [14].

Personality traits have been defined as “relatively stable, consistent, and enduring internal characteristics inferred from an individual’s patterns of behavior, attitudes, emotions, and habits” [15]. In the growth model proposed by Tedeschi and Calhoun [6], personality traits are described as playing a key role in the development of post-traumatic growth. Researchers have also conducted a number of related studies on the relationship between personality traits and posttraumatic growth. Extraversion has been found to positively affect PTG [16], this may be because people who are high in extraversion are more likely to express their emotions and interact with others, which promotes PTG in interpersonal interactions.

However, few studies have analyzed the factors influencing post-traumatic growth in first stroke. Therefore, this study will assess the level of post-traumatic growth in first-time stroke patients and comprehensively analyze their influencing factors on a post-traumatic growth-related model based on post-traumatic growth. It is hoped that this will reduce the negative psychological experience of patients and increase their confidence and hope in their ability to cope with their illness, thus better promoting their recovery.

### Aims

The main objectives of this study included: (1) to describe the posttraumatic growth of patients with first-ever stroke; (2) to investigate the factors influencing posttraumatic growth in patients with first-ever stroke.

## Methods

### Design and sample

The study was a cross-sectional descriptive study [17]. Patient eligibility criteria for inclusion in this study were as follows: (1) patients with a diagnosis of stroke; (2)Age ≥ 18 years; (3) Patients with first stroke; (4) Stable condition, conscious and able to co-operate in completing the scale; (5) Patients and their family members who voluntarily participate in the study with informed consent and sign the informed consent form; Patients diagnosed with other serious life-threatening diseases or recently suffered from other serious trauma or with a history of mental illness are excluded or patients with a history of psychiatric illness were excluded.

The number of participants required for this study was determined using Cohen’s power analysis for multiple linear regression, with a significance level of α = 0.05, a target power of 1 - β = 0.80, an expected medium effect size of f² = 0.15, and eight predictor variables, resulting in a calculated minimum sample size of 109 individuals. To account for a potential dropout rate of 20%, we adjusted the sample size to 137 participants. Ultimately, we recruited 166 participants, which provided a safety margin of 21.2% above the minimum requirement, thereby enhancing the statistical robustness of our study. Post hoc power analysis revealed that the statistical power for detecting a medium effect was 0.959, significantly exceeding the traditional threshold of 0.80, ensuring the reliability and validity of our statistical inferences.

### Data collection

The samples were convenience sampled from two divinity wards in a tertiary care hospital in Shenzhen, Guangdong Province, China. Two postgraduate students, trained in data collection and quality control methods, were responsible for recruiting participants and collecting data. Before distributing the questionnaires, the researchers explained the purpose, significance and precautions of the questionnaires and ensured their anonymity. A total of 170 questionnaires were distributed in this study and 166 valid questionnaires were returned with a validity rate of 97.65%.

### Theoretical framework

There are a number of theoretical models that seek to explain the process of post-traumatic growth [18]. A widely utilized model of posttraumatic growth is the Posttraumatic Growth Model proposed by Tedeschi and Calhoun [6]. The model posits that self-representation, coping styles, social support, rumination, and cognitive processing all exert a significant influence on the process of posttraumatic growth following a stressful event. Another significant model is the Life Crisis and Personal Growth Model [19], which places emphasis on the role of life crises in promoting personal growth and adjustment. This model places emphasis on the role of life crises in promoting personal growth and adaptation. According to this model, the ability of a traumatic event to promote personal growth is influenced by personal factors (e.g., age, level of education), environmental factors (e.g., support from friends and family), event-related factors (e.g., event duration), and cognitive factors. (e.g., event duration), and cognitive assessment/coping strategies [19].

The two models elucidate the role of social support in prompting individuals to seek meaning. In a similar vein, both models elucidate the role of personality variables, such as extraversion and even openness to experience, in promoting PTG. With regard to the denial or avoidance of coping mechanisms, both models posit that such coping is not conducive to positive transformation PTG. The primary distinction between the two models lies in their respective methodologies for predicting the impact of event severity on growth. Schaefer and Moss [19]posit that PTG occurs when an individual survives a poor posttraumatic prognosis, while Tedeschi and Cahoon [6]contend that PTG also arises in cases where the posttraumatic prognosis is poor. In a more general sense, Schaefer and Moss [19]propose that a multitude of factors exert a direct influence on PTG. In contrast, Tedeschi and Cahoon [6]contend that ruminative thinking, social support, and acceptance of coping are the most salient predictive factors for PTG. Tedeschi and Calhoun’s [6]model offers a more comprehensive framework that not only focuses on post-traumatic psychological growth but also emphasizes how individuals can facilitate personal growth by reflecting on and processing traumatic experiences. Therefore, the posttraumatic growth model was selected as the theoretical foundation for this study.

### Measures

Sociodemographic disease information this information was collected by two trained graduate nursing students in accordance with the study protocol.

Demographic data items included include sex, age, monthly household income, marital status, ethnicity, medical payment method, degree of education, personality traits, religious belief, residence, number of children, resident manner, and caregiving situation. Items for disease characteristics included stroke type, number of comorbid chronic diseases, family history of stroke, mRS score upon admission, anxiety, and depression.

Sex is categorized as male or female. Age is divided into the following groups: 18–59 years, 60–69 years, 70–79 years, and 80 years or older. Monthly household income is classified into three brackets: 2,000–5,000 RMB, 5,000–10,000 RMB, and over 10,000 RMB. Marital status includes married, unmarried, divorced, widowed, or other (including separated and cohabiting). Ethnicity is divided into Han and other (referring to ethnic minorities such as Hui and Zhuang). The method of medical payment is categorized as medical insurance, out-of-pocket, or public expense. Educational attainment is classified as junior high school and below, junior high school graduation, high school graduation, university graduation and above, and other (including adult education and self-study examinations). Personality traits are divided into introverted, intermediate, and extroverted types. Religious belief is answered with “yes” or “no”. Place of residence is divided into urban and rural areas. The number of children is categorized as no children, one child, or two or more. Living arrangement can be living with a spouse, living with children, living alone, or other (living with friends, communal living). Caregiving situation is categorized as relatives’ care, professional caregiver care, or other (including no care, friends’ care).

Regarding disease characteristics, stroke type is divided into ischemic stroke and hemorrhagic stroke. The number of comorbid chronic diseases is categorized as less than 2, 2 or more, or other (uncertain chronic diseases). Family history of stroke is divided into present or absent. The mRS score upon admission is categorized into 0–2, 3–4, and 5–6. Anxiety is categorized based on scale scores into 0–4, 5–9, and 10–14. Depression is categorized based on scale scores into 0–4, 5–9, 10–14, and 15–19. All clinical data were cross-verified through electronic medical records and patient self-assessment questionnaires.

Post-traumatic Growth was assessed with the Post-traumatic Growth Inventory (PTGI), which was developed to assess growth-related changes after trauma [5]. The instrument has been translated into Chinese by Wang et al. [20].We have obtained permission from the authors to use their questionnaire for this study(Additional file 1).The PTGI consists of 21 items and five factors, including correlation with other items (seven items), new possibilities (five items), personal strength (four items), appreciation of life (three items), and spiritual change (two items). Ratings are made on a 6-point Likert scale ranging from “0 - I have not experienced this change as a result of the crisis” to “5 - I have experienced this change to a great extent as a result of the crisis.” In the present study, the term ‘crisis’ is employed to denote the trauma associated with a stroke diagnosis. Aggregated item scores were calculated to produce a total score ranging from 0 to 105, with higher scores denoting greater post-traumatic growth. The revised version of the scale had a Cronbach’s α coefficient of 0.90, and the Cronbach’s α coefficients for the dimensions ranged from 0.67 to 0.85.

The Simplified Coping Styles Questionnaire (SCSQ) is a tool designed to assess coping strategies. Xie Y [21] adapted this instrument into a simplified version in China in 1998. The scale consists of two dimensions: positive coping and negative coping, with a total of 20 items. The positive coping dimension includes 12 items, while the negative coping dimension includes 8 items. Responses are scored on a 4-point Likert scale, where 0 indicates “no use,” 1 indicates “occasional use,” 2 indicates “sometimes use,” and 3 indicates “frequent use.” The Cronbach’s α coefficients for the subscales of intrusive rumination and deliberate rumination in domestic stroke patients were 0.910 and 0.856, respectively.

The Social Support Rating Scale (SSRS), developed by Xiao et al. [22], has been widely used to assess the level of social support among Chinese individuals. This scale comprises three dimensions: objective support, subjective support, and utilization of social support, with a total of 10 items. The SSRS includes three types of questions: items 1–4 and 8–10 are single-choice questions, scored from 1 to 4 points based on options A to D, respectively. Item 5 is a five-item scale, scored from 1 to 4 points, ranging from no support to full support, with a total score of 20 points for this item. Items 6 and 7 are multiple-choice questions, with scores assigned based on the number of items selected; no score is given if no items are selected. The maximum score for the entire scale is 66 points, with scores < 22 indicating a low level of social support, 22–44 indicating a medium level, and > 44 indicating a high level. The Cronbach’s α coefficients for each dimension of the scale ranged from 0.825 to 0.849, indicating good internal consistency.

The Chinese Event Related Rumination Inventory(C-ERRI), was used to assess the severity of rumination across two distinct dimensions: intrusive rumination and deliberate rumination. Originally developed by Cann et al. [23] and subsequently revised by Dong C et al. [24], the C-ERRI comprises 20 items scored on a 4-point Likert scale. Scoring is as follows: 0 for “never,” 1 for “sometimes,” 2 for “often,” and 3 for “almost always.” The total score ranges from 0 to 60, with higher scores indicating a greater tendency to engage in rumination. The Cronbach’s α coefficients for the intrusive rumination and deliberate rumination subscales among domestic stroke patients were 0.910 and 0.856, respectively [25].

The Generalized Anxiety Disorder-7 (GAD-7) self-assessment scale (GAD-7) developed by Spitzer’s team [26] in 2006 was used to screen for generalized anxiety and assess its severity by asking patients about their mental mood changes in the last 2 weeks. There are 7 entries in total, using a 4-point scale of 0–3, with a total score range of 0–21, 0–4 no anxiety, 5–9 possible mild anxiety, 10–14 moderate anxiety 15–21 severe anxiety. The Chinese version of the GAD-7 was formed by He X et al. [27] Chinese, with a Cronbach’s α coefficient of 0.898.

The Patients Health Questionnaire-9, (PHQ-9) was used was developed by Spitzer et al. [28] in 1999 based on the diagnostic criteria for depressive episodes and was used to assess depressive symptoms in the last two weeks. The questionnaire consists of nine items on a four-point scale of 0–3, with a total score range of 0–27, and the higher the score, the higher the likelihood of screening for a depressive disorder. scores of 0–4, 5–9, 10–14, 15–19, and 20–27 represent no depression, mild, moderate, moderately severe, and severe depression, respectively [29]. The Chinese version was modified by Bian C et al. [30], and a Cronbach’s α coefficient of 0.8383 was obtained through testing in depressed patients with stroke [31].

### Ethical considerations

This study was approved by the Ethics Committee of Shenzhen Second People’s Hospital (Ethics Number: 2024-360-02PJ). The study was performed in accordance with the Helsinki Declaration. In this study, we used a written informed consent form. The consent form detailed the purpose of the study, the study methods, the rights of the participants (including the right to withdraw at any time), the possible risks/benefits, and the confidentiality of the data. To ensure that participants fully understood the content of the informed consent form, we used simple and easy-to-understand language during the explanation process, which was appropriately adapted to the cultural background of the target population. After participants had read and understood the informed consent form, we further confirmed their understanding by asking questions, ensuring that each participant had a clear understanding of the study information. The questionnaire was completed independently by the participants and was anonymous. To protect the privacy of the participants, a unique code was assigned to each participant. Their sociodemographic and clinical diagnostic information (e.g., name, age, stroke type, etc.) was strictly protected in this manner to ensure data confidentiality and security.

### Statistical analyses

SPSS26.0 Statistical software was used to analyze the data. Prior to analysis, data were preprocessed to identify and address missing values and outliers. Missing data were handled through multiple imputation to ensure the completeness and accuracy of the analysis results. Outliers were identified using box-and-line plots and Z-scores, with decisions to correct or exclude them made on a case-by-case basis. The distribution of Post-Traumatic Growth Inventory (PTGI) scores was confirmed to conform to a normal distribution by the Kolmogorov-Smirnov test, with results expressed as mean ± standard deviation. When the measured data are normally distributed and exhibit homogeneity of variances, parametric statistical methods such as the t-test or Analysis of Variance (ANOVA) should be employed for analysis. In cases where the data do not meet these assumptions, non-parametric tests such as the Mann-Whitney U test or the Kruskal-Wallis H test are recommended, and the Spearman rank correlation coefficient was used for correlation analyses. We performed multicollinearity diagnostics on continuous independent variables, including social support, coping styles, and rumination. Subsequently, variables that were statistically significant in the univariate analysis as well as in the correlation analysis were used as independent variables to identify factors affecting post-traumatic growth through multiple linear regression analysis. Categorical variables were encoded using dummy variables. In the multiple regression analysis, the “enter” method was applied to dummy variables, while the “stepwise” method was used for continuous and ordinal variables to determine the factors influencing PTG in first-time stroke patients. We set the probabilities of F to enter or remove a variable as 0.05 and 0.10, respectively. All statistical tests were two-tailed, with a significance level of *P* < 0.05.

## Results

### Characteristics of the study sample

**Fig. 1:**
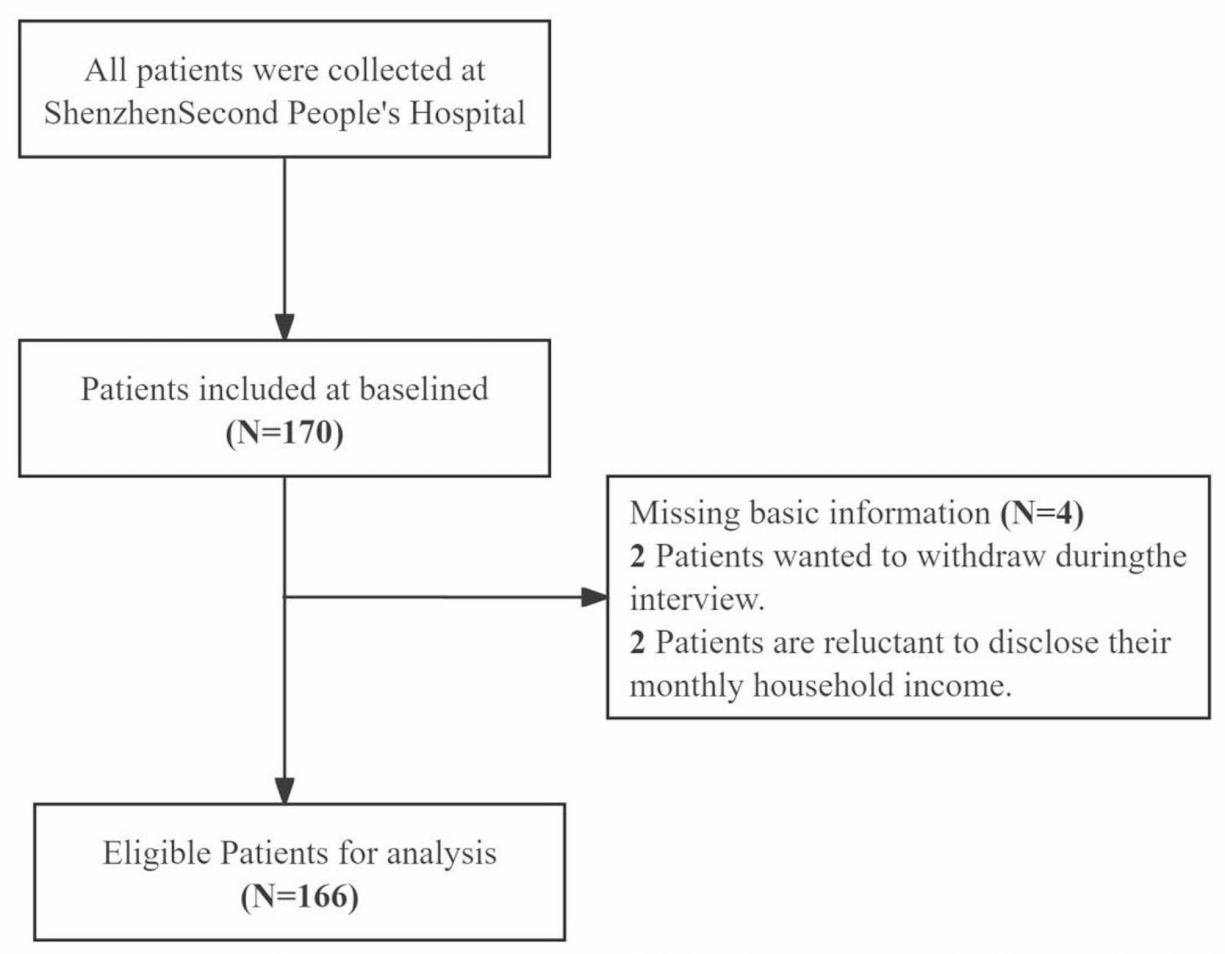
Description of the participant screening process

Initially, 170 patients were enrolled in this study. After excluding 4 cases with missing baseline information, a total of 166 cases were ultimately retained for data analysis. The detailed process is shown in Fig. 1.The percentage of males amounted to 63.9%, and the proportion of females was 36.1%. The age distribution was between 23 and 87 years old (63.21 ± 11.96 years). 47.6% of the patients had a monthly household income between 5,000 and 10,000 yuan, 98.8% were of Han ethnicity, and 85.5% resided in urban areas. In terms of marital status, 81.3% were married, 27.7% had an education level of junior high school or below, 42.8% had an intermediate personality trait, 97% reported no religious beliefs, and 42.2% lived with their spouse. Regarding disease-related data, 94.6% of the patients were diagnosed with ischemic stroke, 45.2% had two or more chronic diseases, and 81.3% had no family history of stroke. Additionally, 59.6% of patients had an mRS score of 0–2, 51.8% showed no anxiety symptoms, and 59.6% were not depressed. Sociodemographic characteristics and disease-related data are detailed in Table 1.

**Table 1:** Participants’ sociodemographic and disease characteristics (*n* = 166)

Variable	Number of examples (*n*)	Composition ratio (%)
**Sex**		
Woman Man	60 106	36.1 63.9
**Age**		
18–59 60–69 70–79 ≥ 80	57 53 49 7	34.3 31.9 29.5 4.2
**Family monthly income**		
2000–5000 RMB 5000–10,000 RMB >10,000 RMB	10 79 77	6.0 47.6 46.4
**Marital status**		
Unmarried Married Dissociation Bereft of one’s spouse Other	7 135 13 10 1	4.2 81.3 7.8 6.0 0.6
**Ethnicity**		
Han ethnic group Other	164 2	98.8 1.2
**Medical payment method**		
Hospitalization insurance At one’s own expense At public expense	147 12 7	88.6 7.2 4.2
**Degree of education**		
Junior high school and below Junior middle school High school College degree or above Other	46 40 44 33 3	27.7 24.1 26.5 19.9 1.8
**Personality traits**		
Introvert type Intermedius type Extravert type	37 71 58	22.3 42.8 34.9
**Religious beliefs**		
Have Not have	5 161	3.0 97
**Residence**		
City Rural area	142 24	85.5 14.5
**Number of children**		
Without children One child Two or more	9 38 119	5.4 22.9 71.7
**Resident manner**		
Living together with a spouse Living together with their children Living alone Other	70 64 26 6	42.2 38.6 15.7 3.6
**Caregiver situation**		
Relatives accompany Professional care Other	136 24 6	81.9 14.5 3.6
**Stroke type**		
Hemorrhagic stroke Ischemic stroke	9 157	5.4 94.6
**type of comorbid chronic disease**		
<2 ≥ 2 Other	67 75 24	40.4 45.2 14.5
**Family history**		
Have Not have	31 135	18.7 81.3
**mRS grade**		
0–2 3–4 5–6	99 64 3	59.6 38.6 1.8
**GAD-7**		
0–4 5–9 10–14	86 65 15	51.8 39.2 9.0
**PHQ-9**		
0–4 5–9 10–14 15–19	99 62 4 1	59.6 36.7 2.4 0.6

### PTG levels and subscale scores for the participant

The total PTGI score was (61.03 ± 13.92). Considering that each scale of the PTGI has a different number of entries, the researchers converted the scores into percentile scores [percentile score = (mean score/highest possible score)*100] in order to compare these values with each other [32]. In the PTGI the subscales with the highest scores were relationship with others, and the lowest subscale was new possibilities (Table 2).

**Table 2:** Scores of dimensions in PTGI (*n* = 166)

Scale	Q50(Q25,Q75)	Scoring range	Score range	Centesimal score
Spiritual change	8(6,11)	0–15	1–14	61
Appreciation of life	13(11,15)	0–30	2–19	66.16
New possibilities	9(7,10)	0–15	3–18	47.78
Relationship with others	19(16,21.3)	0–30	8–28	67.21
Personal strength	12(11,15)	0–20	3–19	66.11

### Relationships between post-traumatic growth, coping strategies, rumination and social support

Correlations between post-traumatic growth, social support, coping strategies, and rumination are displayed in Table 3. PTG was significantly positively correlated with social support, positive coping, and purposeful rumination (*p* < 0.01). Conversely, PTG exhibited a significant negative correlation with negative coping (*p* < 0.01), whereas there was no significant correlation between intrusive rumination and PTG (*p* > 0.05).

**Table 3:** Correlation analysis of post-traumatic growth, social support, coping styles and rumination in first-onset stroke patients

	Post-traumatic growth		Social support	Positive coping	Negative coping	Purposeful rumination	Intrusive rumination
Post-traumatic growth		1					
Social support		0.695**	1				
Positive coping		0.762**	0.616**	1			
Negative coping		− 0.355**	− 0.403**	− 0.227**	1		
Purposeful rumination		0.540**	0.412**	0.558**	− 0.265**	1	
Intrusive rumination		-0.113	− 0.214**	-0.125	0.327**	-0.019	1

**: *p*<0.01

Additionally, we conducted a collinearity diagnosis for the continuous independent variables, including social support, coping strategies, and rumination. The results indicated that the absolute values of the correlation coefficients (r)for all variables were < 0.8, suggesting that there is no collinearity among the independent variables.

### Results of univariate analysis of post-traumatic growth in patients with first-ever stroke

The results of the univariate analysis, as shown in Table 4, indicate that there are statistically significant differences (*P* < 0.05) in post-traumatic growth levels among first-time stroke patients with varying characteristics such as age, monthly household income, education level, ethnicity, personality traits, combined number of chronic diseases, family history, mRS score, anxiety, and depression levels. No significant differences were observed in the remaining factors.

**Table 4:** Results of single factor analysis of post-traumatic growth in patients with first stroke (‾x ± s)

Variable	Mean ± SD	Test value	*P*
**Sex**		-1.360^a^	0.570
Woman Man	(59.08 ± 13.69) (62.13 ± 13.99)		
**Age**		8.138^c^	0.043*
18–59 60–69 70–79 ≥ 80	(65.11 ± 13.82) (59.87 ± 13.21) (57.35 ± 14.34) (62.43 ± 10.10)		
**Family monthly income**		9.899^c^	0.007**
2000–5000 RMB 5000 –10,000 RMB >10,000 RMB	(52.40 ± 11.74) (58.84 ± 14.92) (64.40 ± 12.21)		
**Marital status**		0.813^b^	0.519
Unmarried Married Dissociation Bereft of one’s spouse Other	(55.29 ± 12.50) (61.54 ± 13.29) (60.69 ± 19.39) (60.50 ± 15.67)		
**Ethnicity**		-0.04^a^	0.035*
Han ethnic group Other	(61.02 ± 13.76) (61.50 ± 33.23)		
**Medical payment method**		0.385^b^	0.681
Hospitalization insurance At one’s own expense At public expense	(60.79 ± 13.88) (64.42 ± 13.64) (60.29 ± 16.53)		
**Degree of education**		4.309^b^	0.002**
Junior high school and below Junior middle school High school College degree or above Other	(56.37 ± 14.60) (62.95 ± 13.56) (58.70 ± 12.32) (68.21 ± 12.47) (62.00 ± 17.35)		
**Personality traits**		20.675^b^	0.000**
Introvert type Intermedius type Extravert type	(52.38 ± 12.50) (59.23 ± 12.38) (61.03 ± 13.92)		
**Religious beliefs**		1.082^a^	0.890
Have Not have	54.4 ± 6.23 61.24 ± 1.10		
**Residence**		1.800^a^	0.466
City Rural area	(61.82 ± 14.00) (56.33 ± 12.65)		
**Number of children**		1.057^c^	0.589
Without children One child Two or more	(57.44 ± 14.72) (61.79 ± 14.71) (61.06 ± 13.68)		
**Resident manner**		0.782^c^	0.506
Living together with a spouse Living together with their children Living alone Other	(59.94 ± 12.22) (62.72 ± 14.64) (58.96 ± 15.92) (64.67 ± 16.44)		
**Escort situation**		0.431^b^	0.651
Relatives accompany Professional care Other	(61.44 ± 14.11) (58.58 ± 12.39) (61.50 ± 16.53)		
**Stroke type**		-1.116^a^	0.047*
Hemorrhagic stroke Ischemic stroke	(56.00 ± 7.60) (61.32 ± 14.15)		
**type of comorbid chronic disease**		10.885^c^	0.001**
<2 ≥ 2 Other	(65.85 ± 12.51) (57.57 ± 14.24) (58.38 ± 13.16)		
**Family history**		-0.470^a^	0.374
Have Not have	(59.97 ± 13.61) (61.27 ± 14.02)		
**mRS grade**		11.492^c^	0.003**
0–2 3–4 5–6	(63.71 ± 12.52) (57.05 ± 14.79) (57.67 ± 23.00)		
**GAD-7**		25.994^c^	0.000**
0–4 5–9 10–14	(65.87 ± 14.21) (57.14 ± 10.92) (50.13 ± 13.01)		
**PHQ-9**		28.817^c^	0.000**
0–4 5–9 10–14 15–19	(65.11 ± 14.52) (56.00 ± 9.88) (46.25 ± 7.18)		

Note 1) a: represents T-test; (2)b: represents F-test;(3)c: represents H-test

2) Age: 18–59 (dummy variable); Degree of education: Junior high school and below(dummy variable). Personality traits: Intermedius type(dummy variable)

3)*: *P*<0.05;**P<0.01

### Multivariate analyses of PTG-associated factors

The results of the multivariate linear regression analysis showed that four variables affecting posttraumatic growth in patients with first stroke entered the regression equation: age, positive coping, negative coping, and personality traits, which together explained 64% of the variance. A residual analysis to test the independence of error terms resulted in a Durbin Watson value of 1.934. Because this value was close to 2, the assumption of independence was satisfied. In addition, tolerance was 0.839–0.980, more than 0.1, and VIF was 1.021–1.193, less than 10. Therefore, we are confident that there was no multicollinearity.

Table 5 resents the results of the regression analysis, indicating that positive coping (β coefficient = 0.666) has the strongest positive correlation with post-traumatic growth (PTG) following a first stroke, while negative coping (β coefficient = -0.172) exhibits a negative association with PTG (Table 5). Additionally, extroverted personality traits significantly promote PTG after a first stroke. Regarding age, patients aged 70–79 demonstrate the lowest levels of PTG.

**Table 5:** Multifactor analysis

Variable	Unstandardized coefficients Beta		Std.erro	Standardized beta coefficient	*t*	*p*	95%CI for beta	tolerance	VIF
Constant		42.342	2.508		16.884	0.000**	37.390,47.294		
Age_70–79		-3.539	1.445	-0.116	-2.450	0.015*	-6.392, -0.686	0.980	1.021
Personality traits Extravert type		3.944	1.494	0.136	2.640	0.009**	0.993,6.894	0.839	1.193
Positive Coping		1.374	0.105	0.666	13.134	0.000**	1.167,1.581	0.859	1.165
Negative coping		-0.516	0.150	-0.172	-3.436	0.001**	-0.812, -0.219	0.886	1.129

*R* = 0.80, Adjusted R^2^ = 0.64, F = 72.86 .*P*<0.001

**p<0.01,*p<0.05

## Discussion

### PTG levels in first-time stroke patients

The findings of this study indicate that the average score on the PTGI among first-time stroke patients is 61.03 ± 13.92, suggesting that these patients may have experienced a certain degree of positive psychological change. Given that the maximum possible score on the PTGI is 105, this result could imply that there is still room for improvement in post-traumatic growth among first-time stroke patients. Previous studies have mostly focused on the assessment of post-traumatic growth in cancer patients [33–35], and the results were slightly lower than those in this study. This is related to many factors such as disease characteristics, demographic data and measurement tools of the population included.

Due to the varying number of items and score ranges across different subscales of the PTGI, we used percentile scores to compare the scores within each subscale. In this study, the highest scores were observed in the “Relationship with Others” subscale, followed by “Appreciation of Life” and “Personal Strength”. This may be associated with the emotional relief and interpersonal communication that patients experience after illness due to the care and support from friends and family, indicating that the occurrence of the disease may have a certain facilitative effect on enhancing interpersonal relationships. In contrast, the lowest scores were found in the “Spiritual Change” and “New Possibilities” dimensions. The dimension of new possibilities primarily involves the exploration of new goals, interests, and roles in life for patients. For patients experiencing their first stroke, the onset of the disease is abrupt and unpredictable, which necessitates a psychological transition from shock to adaptation.

### Coping styles are correlated with post-traumatic growth

Coping strategies refer to the relatively stable behavioral and cognitive methods that individuals employ when facing various types of stress. The results of this study indicate a significant positive correlation between positive coping and post-traumatic growth scores among first-time stroke patients, suggesting that positive coping is an important influencing factor for PTG. This is consistent with the research by Bellizzi et al. [36], which found a high positive correlation between positive coping strategies and PTG, and demonstrated that positive coping significantly predicts growth. In contrast, negative coping is significantly negatively correlated with the PTG scores of first-time stroke patients. As noted by Kolokotroni et al. [37], when patients confront illness, adopting a correct cognition and positive coping strategies enable them to face the trauma directly, leading to a higher level of PTG. Conversely, patients with more negative coping styles tend to respond pessimistically and may even succumb or evade, resulting in adverse outcomes. Different coping strategies exert positive or negative influences on patients, leading to distinct outcomes. Therefore, there is an urgent need to advocate for more research focusing on patients’ positive coping strategies, with the aim of exploring and enhancing these strategies to elevate their PTG levels, thereby improving their quality of life and psychological well-being.

### Sociodemographic contribute to post-traumatic growth

Our study results indicate that there are differences in post-traumatic growth levels across age and personality traits. Significant differences in PTG levels were observed among first-time stroke patients of different ages, with younger patients exhibiting notably higher PTG levels, which is consistent with previous research [33, 38].Compared to older first-time stroke patients, younger individuals are more likely to re-evaluate their initial perceptions of traumatic events. When confronted with overwhelming situations that challenge their understanding of trauma, younger patients often reassess and acknowledge the trauma, leading to an increase in PTG [39]. In contrast, older first-time stroke patients may experience lower PTG levels due to a reduced optimism about maintaining an active lifestyle.

In addition, there were statistically significant differences in PTGI scores between patients with different personality characteristics at first stroke. Notably, patients with extroverted personality at first stroke had significantly higher post-traumatic growth scores than those with introverted or intermediate personality (*P* < 0.05). This discovery is consistent with the results of studies by [40]and [41], suggesting that one possible interpretation of this outcome is based on how extraverts are more optimistic. Specifically, optimistic individuals tend to focus on the most important matters, reject unrealistic goals that are inconsistent with reality, which may lead to post-traumatic growth [42]. Furthermore, individuals with high extroversion are more likely to employ a greater range of support-seeking, problem-solving, and cognitive reframing strategies, achieving higher levels of PTG [43].

### Strengths

Although the majority of the findings have previously been reported in stroke patients, this study has several noteworthy strengths. Primarily, it provides a unique opportunity to synthesis the factors influencing post-traumatic growth. It conducts an integrative exploration of the influences on post-traumatic growth, including personal, environmental, and event-related factors. Secondly, the results of this study provide empirical evidence for a functional model of post-traumatic growth.

### Limitations

This study has several limitations. Firstly, the study utilized a convenience sampling method to select participants from a tertiary hospital in Shenzhen, among whom 94.6% had ischemic stroke, which may affect the generalizability of the research findings. According to the World Stroke Organization (WSO) report in 2022, ischemic stroke accounts for up to 87% of all stroke cases, aligning with our findings. Future research should expand the sample coverage to include a broader range of stroke patients. Secondly, the cross-sectional design of this study makes it difficult to accurately establish causal relationships between variables. Therefore, longitudinal studies are needed to track data over the long term for each patient to more precisely grasp the trends in these variables.

### Implications for practice and future study

Based on these findings, we propose several practical recommendations. Firstly, the positive correlation between extroverted personality traits and post-traumatic growth can assist clinical healthcare providers in identifying patients who may experience lower levels of PTG. Secondly, personalized intervention strategies should be tailored according to patients’ personality traits. For introverted patients, encouraging participation in social activities and group therapy, actively organizing patient panel discussions, and selecting extroverted patients as peer educators to share their experiences can create a positive atmosphere that inspires and motivates introverted patients, thereby promoting an overall increase in PTG levels. Concurrently, nursing practices should incorporate elements that foster positive coping strategies, such as cognitive restructuring and problem-solving skills, as positive coping is also positively correlated with PTG. For patients exhibiting negative coping strategies, additional support and interventions, such as psychological education and behavioral therapy, should be provided to help them develop healthier coping mechanisms. Furthermore, given that older patients may face more challenges in PTG, this group requires special attention, with targeted support and resources being provided.

## Conclusion

First-time stroke patients experience positive changes after a traumatic event. The findings suggest that extraverted, positively coping first-time stroke patients tend to experience more posttraumatic growth, and conversely, negative coping has a negative impact on promoting posttraumatic growth. This suggests that we should pay attention to patients’ coping styles in clinical practice, guide them to adopt positive coping strategies through counseling and other means, and provide more psychological support for introverted and older patients. These findings not only help healthcare professionals better identify patient groups with potential for post-traumatic growth, but also provide a theoretical basis for developing individualized psychological intervention programs. Future studies could further explore how to design more targeted interventions based on patients’ personality traits to maximize the potential for promoting posttraumatic growth. These meaningful findings provide several new opportunities to promote posttraumatic growth in first-time stroke patients.

### Clinical practice relevance

This study reveals the positive changes in first-time stroke patients after experiencing a traumatic event, further confirming that post-traumatic growth occurs not only in cancer patients, but also exists in patients with chronic diseases, suggesting that clinical staff should pay more attention to the psychological changes of patients from a positive perspective. Based on the functional model of post-traumatic growth, this study investigated the influencing factors of post-traumatic growth in patients with incipient stroke. It was found that extraversion and positive coping styles were important factors in promoting posttraumatic growth, and healthcare professionals should pay attention to patients’ coping styles and personality traits, provide more psychological support for introverted patients, and guide patients to adopt positive coping strategies. These targeted interventions can promote patient recovery and are important for improving clinical outcomes.

## Supplementary Information

Below is the link to the electronic supplementary material.

**Supplementary Material 1**: **Additional file 1**: Agreement on the Use of Assessment Tools “Simplified Chinese version of Post-traumatic Growth Questionnaire”.

## Data Availability

The data analyzed in this study involve the personal information of patients. Although we have done anonymous processing, the data are not disclosed for better protection of patients’ privacy, but can be obtained from the corresponding author upon reasonable request.

## Abbreviations

GBD: Global burden of disease
CVA: Cerebrovascular accident
PTG: Posttraumatic growth
PTGI: Post-traumatic growth inventory
SSRS: Social support rating scale
SCSQS: Simplified coping styles questionnaire
C-ERRI: Chinese event related rumination inventory
GAD-7: Generalized anxiety disorder-7
PHQ-9: Patients health questionnaire-9
SPSS: Statistical package for the social sciences
P25: 25th percentile
P50: 50th percentile
P75: 75th percentile
