# Supplementary material for: Post-traumatic growth and its influencing factors in first-episode stroke patients: a cross-sectional study
Source: BMC Psychol. 2025 Aug 27;13:973. doi: 10.1186/s40359-025-03347-y (PMC12392602; doi:10.1186/s40359-025-03347-y)
Supplement: Supplementary file 1 — Supplementary Material 1: Additional file 1: Agreement on the Use of Assessment Tools “Simplified Chinese version of Post-traumatic Growth Questionnaire”. [file 40359_2025_3347_MOESM1_ESM.docx]

**Agreement on the use of “Simplified Chinese Post-Traumatic Growth Questionnaire”**

Party A: Liu Xiaohong

Party B: Hu Minli

Through negotiation, both parties agree that the following terms shall be observed when using “the Simplified Chinese Version of Post-traumatic Growth Questionnaire”:

1. Party A agrees that Party B shall use “the Simplified Chinese version of Post-traumatic Growth Questionnaire”free of charge.

2. Party B shall provide the results of the application evaluation tool to Party A free of charge, including the use of the object, the number of people, the results obtained (including the result of each question for each person and the total score), and the opinions and suggestions on the questionnaire used.

3.The evaluation results and other data provided by Party B to Party A shall only be used by Party A for questionnaire modification and related research, and shall not be published separately without party B's consent.

4. Party B shall maintain confidentiality of the test items in this assessment tool and shall not transfer the assessment tool to others for use in any form. If Party B needs to reuse the assessment tool, it must obtain Party A's consent. In case Party B transfers the assessment tool in whole or in part to others for use, reuse, or disclosure of test items without authorization, Party A shall hold Party B legally liable.

5. Any paper or achievement formed by Party B after using “the Simplified Chinese version of the Post-traumatic Growth Questionnaire”shall be published after communication and negotiation with Party A, and shall indicate the development unit and developer of the tool, and provide the electronic version of the paper to Party A.

Party A: Liu Xiaohong Party B: Hu Minli


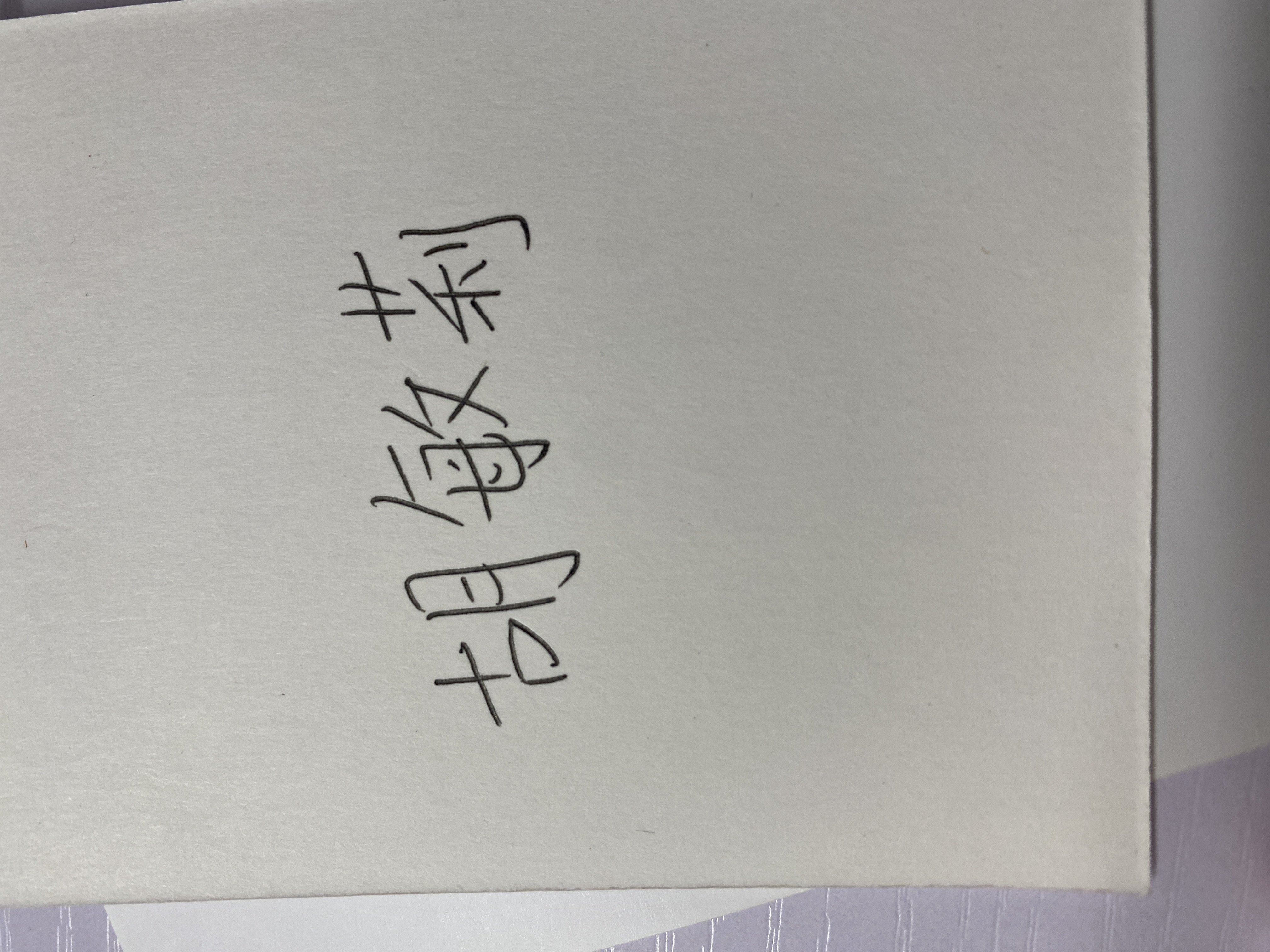
Signature of the representative:
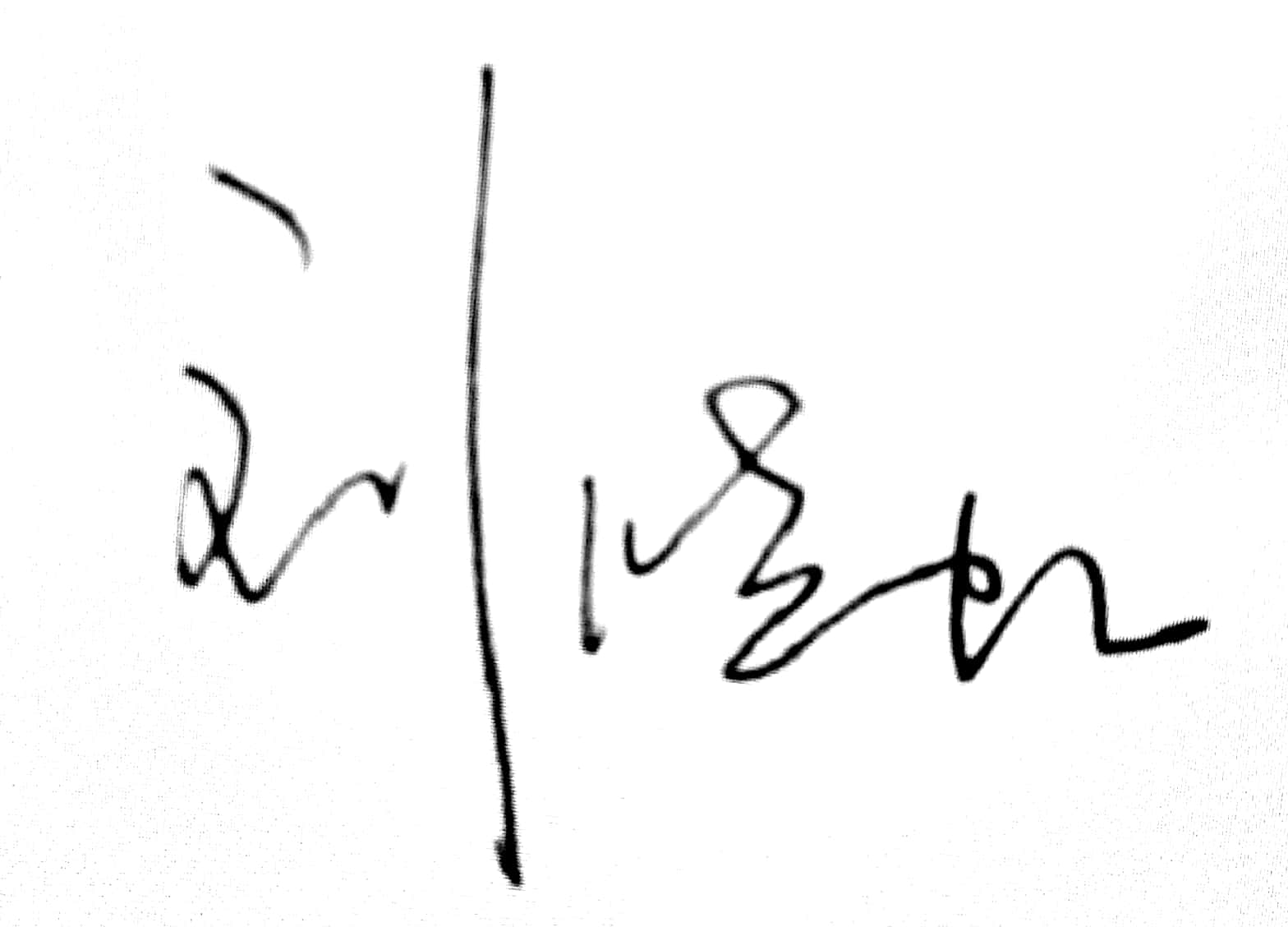
Signature of the representative:

May 28,2025 May 28,2025

The Simplified Chinese Version of the Trauma Post-Growth Inventory (C-PTGI) was developed by Professor Tedeschi and his team. The nursing psychology research team at the Second Military Medical University introduced the English version to mainland China, subsequently localized, revised, and culturally adapted into a simplified Chinese version for post-traumatic growth assessment in trauma populations. This self-report scale comprises 20 items across five dimensions. The consistency reliability coefficients (Cronbach's α) range from 0.611 to 0.874, demonstrating excellent psychometric properties suitable for post-traumatic growth studies among trauma patients in mainland China. It can also be applied to other injury populations.

Simplified Chinese version of Post-traumatic Growth Questionnaire

Note: There are 20 questions in the table, which ask you about the changes that this accident may bring to you. Please read each sentence carefully, and then choose the answer that is closest to your situation for each question and check it with a "√".

| 1 | I changed the order of important things in my life | 11 | I could have done better with my life |
| --- | --- | --- | --- |
| 2 | I have a greater understanding of the value of my life | 12 | I'm more comfortable with the end result of anything |
| 3 | I developed new interests | 13 | I can cherish every day more |
| 4 | I feel more dependent on myself | 14 | This incident has brought me a new opportunity |
| 5 | I have a better understanding of the spiritual level | 15 | I have more compassion for others |
| 6 | I know I can count on others when I'm in trouble | 16 | I put more energy into relationships |
| 7 | I set a new path for my life | 17 | I prefer to change things that need to be changed |
| 8 | I feel more connected to others | 18 | I found myself stronger than I thought |
| 9 | I prefer to express my feelings. | 19 | I have a deeper understanding of how beautiful the world is |
| 10 | I know I can handle the difficulties better | 20 | I'm more comfortable with the idea that I need others |

The C-PTGI is a self-report instrument designed for participants to complete independently. When administering the questionnaire, individuals must carefully read and fully understand the instructions before completing each item. The assessment employs a Likert 6-point scale (0-5 points), where responses range from "experiencing no post-traumatic change" to "experiencing significant post-traumatic changes," with total scores ranging from 0 to 100. Higher scores indicate greater levels of post-traumatic growth.

The items included in each factor are as follows:

1. Life Enlightenment Factors: 6 items including 2nd, 5th, 11th, 13th, 15th, and 19th.
2. Personal Power Factors: 3 items covering 10th, 12th, and 18th.
3. New Possibilities Factors: 4 items spanning 9th, 14th, 16th, and 17th.
4. Interpersonal Relationships Factors: 3 items comprising 6th, 8th, and 20th.
5. Self-Transformation Factors: 4 items including 1st, 3rd, 4th, and 7th.
